# Comparing symptoms, treatment patterns, and quality of life of ankylosing spondylitis and non-radiographic axial spondyloarthritis patients in the USA: findings from a patient and rheumatologist Survey

**DOI:** 10.1007/s10067-021-05642-6

**Published:** 2021-02-20

**Authors:** Theresa Hunter, David Sandoval, Nicola Booth, Elizabeth Holdsworth, Atul Deodhar

**Affiliations:** 1 Eli Lilly and Company, Indianapolis, IN USA; 2 Adelphi Real World, Bollington, Cheshire, UK; 3 Oregon Health & Science University, Portland, OR USA

**Keywords:** Ankylosing spondylitis, Non-radiographic axial spondyloarthritis, Quality of life, Treatment patterns

## Abstract

**Objectives:**

The aim of this study was to compare the symptoms, treatment patterns, and quality of life (QoL) of ankylosing spondylitis (AS) patients to non-radiographic axial spondyloarthritis (nr-axSpA) patients in the USA.

**Method:**

A cross-sectional survey was conducted with rheumatologists and their consulting patients in the USA from June through August 2018. Patients who had a rheumatologist confirmed diagnosis of AS and nr-axSpA were eligible to participate. Patient demographics, symptoms, and medication use were reported by the rheumatologist, while work disability and QoL measures were reported by the patient. Patient demographics, symptoms, QoL and treatment patterns of AS and nr-axSpA patients were compared using parametric tests and non-parametric tests when appropriate.

**Results:**

A total of 515 AS patients and 495 nr-axSpA patients were included in this analysis. A higher proportion of AS patients were male (*p* < 0.001), older (*p* = 0.014), and more likely to be prescribed a biologic (*p* < 0.0001). On average, AS patients experienced slightly more symptoms at diagnosis (*p* = 0.023); however, nr-axSpA patients were more likely to experience enthesitis (*p* = 0.048) and synovitis (*p* = 0.003). Patient reported outcomes such as the ASAS Health Index (*p* = 0.171), ASQoL (*p* = 0.296), BASDAI (*p* = 0.124), and WPAI (*p* = 0.183) were similar between AS and nr-axSpA patients after adjusting for confounding variables such as medication use.

**Conclusions:**

AS and nr-axSpA patients share the same clinical features. The burden of the disease, as assessed by QoL measurements, is also similar in AS and nr-axSpA patients; however, despite these similarities, patients with nr-axSpA are less likely to be treated with a biologic.

****Key Points**:**

**Table:** 

*• Ankylosing spondylitis and non* *• radiographic axial spondyloarthritis patients share similar clinical features and burden of disease.* *• Quality of life is similar among ankylosing spondylitis and non* *• radiographic axial spondyloarthritis after adjusting for current treatment patterns.*

## Introduction

Axial spondyloarthritis (axSpA) is a chronic inflammatory disease that mainly affects the axial skeleton and sacroiliac joints [1]. It is estimated that up to 1.4% of the adult population in the USA have axSpA [2]. AxSpA is an umbrella term that includes patients with ankylosing spondylitis (AS) [3] and non-radiographic axial spondyloarthritis (nr-axSpA) [4, 5]. Many rheumatologists and professional organizations, such as ASAS and Spondyloarthritis Research and Treatment Network (SPARTAN), consider AS and nr-axSpA to be part of one disease spectrum (axSpA) [6, 7]. AS in its most advanced expression can be characterized by severe spinal immobility and functional disability caused by fusion of the spine [8]. Patients with nr-axSpA can sometimes progress to AS; however, not all patients with nr-axSpA progress to AS [8]. Progression from nr-axSpA to AS has been reported to occur in approximately 5% to 12% of patients after 2 years [9, 10] and approximately 25% of patients after 15 years [11].

Differentiating AS and nr-axSpA based on symptoms, disease activity, function, and quality of life (QoL) may not be possible, as studies have shown many similarities between these two groups [12–14]. Studies comparing AS and nr-axSpA patients have predominately been conducted outside of the USA [12, 15, 16]. In this study, we compare the symptoms, treatment patterns, and patient-reported outcomes (PROs) of AS patients and nr-axSpA in the USA in a real-world setting.

## Materials and methods

### Study design and study population

A cross-sectional survey was conducted with rheumatologists and their patients in the USA from June through August 2018. The survey methodology was implemented as previously published [17] and adapted to the AS and nr-axSpA population. Rheumatologists seeing at least 10 AS and nr-axSpA patients in a typical month were eligible to participate in this cross-sectional survey.

A geographically representative sample of eligible rheumatologists (*n* = 88) in the USA was included in this study and completed patient record forms for the next ten consecutive axSpA patients (5 AS and 5 nr-axSpA). The diagnosis of AS or nr-axSpA was made by the clinical judgement of the rheumatologist. The rheumatologist completed the patient record forms which included patient demographics, disease status, remission status, clinical characteristics, and current medication use. Presence of symptoms were recorded by the rheumatologist by selecting the symptoms from a list provided.

AS and nr-axSpA patients were invited to complete a survey independent of their rheumatologist. As part of the survey, AS and nr-axSpA patients were asked to complete a patient declaration page where they agreed to complete the survey in accordance with the Health Insurance Portability and Accountability Act (HIPAA). Patients provided consent for de-identified and aggregated reporting of research findings. Data were de-identified according to HIPAA regulations before receipt by Adelphi Real World. All questionnaires used in the survey were reviewed and approved by Western Institutional Review Board.

### Patient-reported outcomes

Disease activity was measured using the Bath Ankylosing Spondylitis Disease Activity Index (BASDAI) [18, 19]. Health-related quality of life (HRQoL) was assessed using the following patient-reported outcome measures: Assessment of SpondyloArthritis international Society Health Index (ASAS HI) [20], Ankylosing Spondylitis Quality of Life (ASQoL) [21], and the European Quality of Life-5 Dimensions-5 Level (EQ-5D-5L) Visual Analog Scale (VAS) [22]. Work productivity and impact of axSpA on activity impairment outside of work was measured by the Work Productivity and Activity Impairment (WPAI) questionnaire [23].

### Statistical analyses

Descriptive analyses were conducted for the entire axSpA population and then stratified by AS and nr-axSpA patients. Summary statistics were used to compare patient demographics, clinical characteristics, treatment patterns, and PROs between AS and nr-axSpA patients. Categorical variables were analyzed by frequency counts and percentages, with Chi-square tests used for subgroup analyses. Continuous variables were analyzed by mean (standard deviation [SD]), with two-sample *t*-tests used for subgroup analyses. In addition, ordinary least squared regressions were performed on the PRO variables after adjusting for confounders such as age, sex, body mass index (BMI), Charlson Comorbidity Index (CCI), overall severity, and treatment. Marginal means were calculated based on the regression model.

## Results

### Demographics

A total of 1010 axSpA patients (AS, 515; nr-axSpA, 495) were included in this analysis; 570 axSpA patients completed the patient survey (AS, 284; nr-axSpA, 286). Demographic information for all axSpA patients, as well as those with AS and nr-axSpA, is included in Table 1. Overall, 62.5% (*n* = 631) of axSpA patients were male, had a mean age of 45.2 years, mean BMI of 27.3, and 77.7% were employed either full-time or part-time. A statistically higher proportion of AS patients were male (71.3% vs. 53.3%; *p* < 0.001) and older (mean age: 46.3 vs. 44.2; *p* = 0.014) when compared to nr-axSpA patients (Table 2).

**Table 1:** Patient-reported outcomes

PRO measure	Concepts measured	Number of items	Response options	Score ranges
BASDAI^18-19^	Disease activity including • Fatigue • Spinal pain • Peripheral arthritis • Enthesitis • Intensity of morning stiffness • Duration of morning stiffness	6	0–10 numeric rating scale	0–10 (“no disease activity” to “high disease activity”)
ASAS HI^20^	Overall health and functioning	17	“I agree” (score 1), “I do not agree” (score 0), or “not applicable” (only for item 7 and 8).	0–17 (“good health” to “poor health”)
ASQoL^21^	Quality of life	18	“Yes (score 1)” or “No (score 0)”	0–18 (“good QoL” to “poor QoL”)
EQ-5D VAS^22^	Overall health status	0–100	0–100 visual analogue scale	0–100 (“the worst health you can imagine” to “the best health you can imagine”)
WPAI-SpA^23^	• Absenteeism • Presenteeism • Overall work impairment • Activity impairment	6	0–10 numeric rating scale	• Percentage of Absenteeism (0–100%) • Percentage of Presenteeism (0–100%) • Overall work impairment score (combining absenteeism and presenteeism) (0–100%) • Percentage of activity impairment (0–100%)

**Table 2:** Patient demographics

	axSpA patients *N* = 1010	AS patients *N* = 515	nr-axSpA patients *N* = 495	*p* Value
Sex				< 0.001
Male	631 (62.5%)	367 (71.3%)	264 (53.3%)	
Female	379 (37.5%)	148 (28.7%)	231 (46.7%)	
Age, mean	45.2	46.3	44.2	0.014
Ethnic, origin				0.420
White/Caucasian	811 (80.3%)	417 (81.0%)	394 (79.6%)	
African American	77 (7.6%)	43 (8.3%)	34 (6.9%)	
Native American	3 (0.3%)	1 (0.2%)	2 (0.4%)	
Asian	31 (3.1%)	18 (3.5%)	13 (2.6%)	
Middle Eastern	8 (0.8%)	3 (0.6%)	5 (1.0%)	
Mixed race	19 (1.9%)	7 (1.4%)	12 (2.4%)	
Other	1 (0.1%)	1 (0.2%)	0 (0.0%)	
Hispanic/Latino	60 (5.9%)	25 (4.9%)	35 (7.1%)	
BMI (kg/m^2^), mean	27.3	27.5	27.1	0.185
Smoking status*				0.634
Current smoker	101 (10.8%)	48 (10.1%)	53 (11.6%)	
Ex-smoker	195 (20.9%)	104 (21.8%)	91 (19.9%)	
Never smoked	638 (68.3%)	325 (68.1%)	313 (68.5%)	
Employment status**				0.112
Full-time	708 (70.5%)	364 (71.2%)	344 (69.8%)	
Part-time	72 (7.2%)	33 (6.5%)	39 (7.9%)	
Homemaker	57 (5.7%)	20 (3.9%)	37 (7.5%)	
Student	26 (2.6%)	12 (2.3%)	14 (2.8%)	
Unemployed	43 (4.3%)	23 (4.5%)	20 (4.1%)	
Retired	87 (8.7%)	52 (10.2%)	35 (7.1%)	
Long-term sick leave	11 (1.1%)	7 (1.4%)	4 (0.8%)	
Disease status				0.484
Improving	298 (29.5%)	145 (28.2%)	153 (30.9%)	
Stable	557 (55.1%)	282 (54.8%)	275 (55.6%)	
Unstable	87 (8.6%)	47 (9.1%)	40 (8.1%)	
Deteriorating	66 (6.7%)	41 (8.0%)	27 (5.5%)	
In remission	390 (41.5%)	201 (42.2%)	189 (40.7%)	0.644
Rheumatologist’s global assessment VAS***, mean	31.8	32.4	31.1	0.744
Charlson Comorbidity Index, mean	0.15	0.10	0.20	0.222
Joint inflammation or stiffness	336 (33.3%)	156 (30.3%)	180 (36.4%)	0.045
Inflammatory back pain	432 (42.8%)	215 (41.7%)	217 (43.8%)	0.525
Morning stiffness for more than 30 min	372 (36.8%)	193 (37.5%)	179 (36.2%)	0.696
HLA B27 positive at diagnosis	504 (54.3%)	270 (57.9%)	234 (50.6%)	0.030
Alternating buttock pain	65 (6.4%)	33 (6.4%)	32 (6.5%)	1.000
Dactylitis	27 (2.7%)	13 (2.5%)	14 (2.8%)	0.846
Enthesitis	76 (7.5%)	34 (6.6%)	42 (8.5%)	0.284
Tendonitis	81 (8.0%)	45 (8.7%)	36 (7.3%)	0.419
Synovitis	69 (6.8%)	31 (6.0%)	38 (7.7%)	0.320
Arthritis	176 (17.4%)	92 (17.9%)	84 (17.0%)	0.740
Osteoporosis of the spine	31 (3.1%)	24 (4.7%)	7 (1.4%)	0.003

*Smoking status: axSpA, *n* = 934; AS, *n* = 477; nr-axSpA *n* = 457

**Employment Status: axSpA, *n* = 1004; AS, *n* = 511; nr-axSpA, *n* = 493

***Physician’s Global Assessment: axSpA, *n* = 160; AS, *n* = 85; nr-axSpA, *n* = 75

### Clinical characteristics and spondyloarthritis features

The clinical characteristics and extra-articular manifestations were similar between AS and nr-axSpA patients (Table 2). At the time of diagnosis, nr-axSpA patients were more likely than AS patients to have enthesitis (*p* = 0.048) and synovitis (*p* = 0.003). At the time of diagnosis, AS patients were more likely to have osteoporosis of the spine (*p* = 0.021) and elevated CRP (*p* = 0.022) and were HLA-B27 positive (*p* = 0.030) when compared to nr-axSpA patients.

### Disease status and disease activity

The majority of axSpA patients’ current disease status was reported by their rheumatologist as stable or improving (84.6%). AS and nr-axSpA patients’ current disease status (*p* = 0.484) and remission rates were similar (42.2% vs. 40.7%; *p* = 0.644). The mean scores of the physician’s global assessment (PGA) in AS and nr-axSpA patients were comparable (32.4 vs. 31.1; *p* = 0.745). The mean score of the patient’s global assessment (PtGA) was 34.4 for AS and 31.5 for nr-axSpA patients, which was not statistically different (*p* = 0.447). Disease activity, as measured by the mean BASDAI, was also similar between AS and nr-axSpA patients (*p* = 0.124).

### Quality of life

PROs measuring overall function, health, and quality of life such as the ASAS HI, ASQoL, and EQ-5D VAS were similar between AS and nr-axSpA patients (Table 3). The mean ASAS HI score was 5.7 for AS patients and 5.2 for nr-axSpA patients (*p* = 0.171). The mean ASQoL score was 6.3 for AS patients and 5.8 for nr-axSpA patients (*p* = 0.296). The mean EQ-5D VAS was 75.7 for AS patients and 74.9 for nr-axSpA patients (*p* = 0.590). After adjusting for confounding variables, the marginal means of the ASAS HI, ASQoL, and EQ-5D VAS were similar between AS and nr-axSpA patients (Table 3).

**Table 3:** Mean and marginal means of patient reported outcome measures

		Means			Marginal means*		
	Questionnaire score range	AS	nr-axSpA	*p* Value	AS	nr-axSpA	*p* Value
ASAS Health Index	0–17	5.7 (*n* = 274)	5.2 (*n* = 276)	0.171	5.7	5.3	0.295
ASQoL	0–18	6.3 (*n* = 272)	5.8 (*n* = 273)	0.296	6.2	5.9	0.383
BASDAI	0–10	3.2 (*n* = 276)	2.9 (*n* = 276)	0.124	3.1	3.0	0.414
EQ-5D VAS	0–100	75.7 (*n* = 277)	74.9 (*n* = 276)	0.590	76.4	74.2	0.122
Patient’s global assessment VAS	0–100	34.4 (*n* = 86)	31.5 (*n* = 77)	0.447	33.7	32.2	0.685
WPAI							
Absenteeism	0–100	5.0 (*n* = 174)	4.0 (*n* = 178)	0.579	4.9	4.1	0.641
Presenteeism	0–100	21.3 (*n* = 189)	21.9 (*n* = 188)	0.749	21.9	21.2	0.655
Overall work impairment	0–100	23.1 (*n* = 170)	23.6 (*n* = 175)	0.788	23.8	22.9	0.663
Activity impairment	0–100	30.2 (*n* = 269)	27.6 (*n* = 271)	0.183	29.2	28.6	0.743

*Marginal means were calculated from the ordinary least squared regression models after adjusting for age, sex, BMI, Charlson Comorbidity Index, overall severity, and treatment

### Work Productivity and Activity Impairment

The majority of AS (*n* = 364; 71.2%) and nr-axSpA (*n* = 344; 69.8%) patients reported working full-time at the time of the survey. An additional 6.5% (*n* = 33) of AS patients and 7.9% (*n* = 39) of nr-axSpA patients indicated that they worked part-time at the time of the survey. Mean rates of absenteeism (*p* = 0.579), presenteeism (*p* = 0.749), work productivity (*p* = 0.788), and activity impairment (*p* = 0.183), as assessed by the WPAI questionnaire, were similar between AS and nr-axSpA patients (Table 3). These results did not differ when stratified by treatment. After adjusting for confounding variables, the marginal means of absenteeism, presenteeism, work productivity, and activity impairment were similar between AS and nr-axSpA patients (Table 3).

### Medication use

**Fig. 1:**
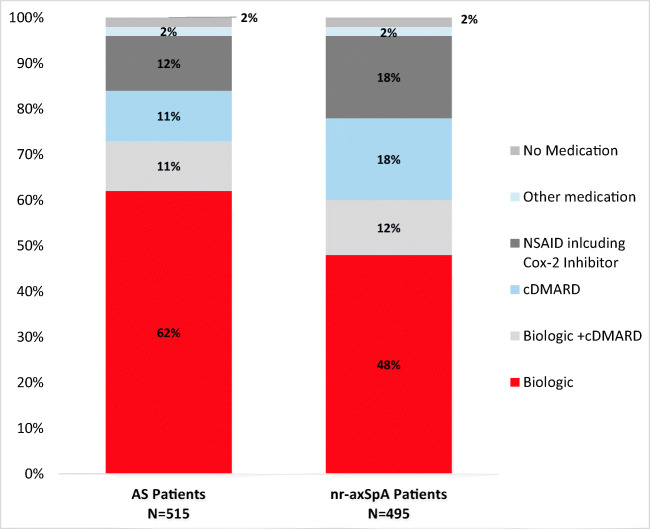
Current medication use

Two-thirds (66.7%) of axSpA patients were currently receiving a biologic. Overall, 55.3% were receiving a biologic as monotherapy, and 11.4% were receiving a biologic in combination with a conventional disease-modifying antirheumatic drug (cDMARD) (Fig. 1). AS patients were more likely to receive a biologic than nr-axSpA patients (73.6% vs. 59.6%, *p* < 0.001). Nr-axSpA patients were more likely to be prescribed a cDMARD (18.4% vs. 11.1%) or nonsteroidal anti-inflammatory drug (NSAID)/Cox-2 Inhibitor (18.2% vs. 11.8%) than AS patients.

## Discussion

This analysis of a large real-world survey of rheumatologists and their axSpA patients provides a comparison of AS and nr-axSpA patients in the USA. This analysis was conducted to compare clinical and demographic characteristics, disease activity, HRQoL, work impairment, and treatment patterns of AS and nr-axSpA patients. Similar to previous studies, our analysis showed that AS and nr-axSpA patients share many clinical symptoms and experience a similar burden of disease [12–14, 24]. The most important finding of our study is that despite the similar burden of disease, patients with nr-axSpA are receiving biologics less commonly than AS patients. Over two-thirds of AS patients were prescribed biologic therapy either as a monotherapy or in combination with a cDMARD. In comparison, only 59.6% of nr-axSpA patients were prescribed biologic therapy. Over one-third of nr-axSpA patients were prescribed a cDMARD or NSAID compared to only one-fourth of AS patients.

Consistent with previous literature, AS patients in our study were more likely to be male, older, and working full-time in comparison to nr-axSpA patients [12, 13, 15, 25]. The PtGA scores, PGA scores, current pain levels, and QoL were similar between AS and nr-axSpA patients. These findings are comparable to those found in the German Spondyloarthritis Inception Cohort (GESPIC), which reported no significant difference between PtGA and total pain score of AS and nr-axSpA patients [14].

Overall, there were few statistically significant differences in the clinical characteristics and symptoms reported by AS and nr-axSpA patients in our study. Patients with AS were more likely to be HLA-B27 positive when compared to nr-axSpA patients; however, the rates of HLA-B27 positivity among AS patients in our study (57.6%) was lower than that in previous research. In the Spanish REGISPONSER database, they reported that 83% of AS patients were HLA-B27 positive [26]. Our study also found that many AS and nr-axSpA patients have concomitant peripheral disease. Rates of arthritis, tendonitis, and dactylitis were similar between AS and nr-axSpA patients. Similar to the GESPIC cohort [14], AS patients in our study were more likely to have loss of movement and osteoporosis of the spine when compared to nr-axSpA patients. The more compromised function of patients with AS may be attributed to the structural changes in the spine [27].

The efficacy of anti-tumor necrosis factor (TNF) treatment in nr-axSpA has been shown to be similar to that in AS, specifically in patients with objective signs of inflammation at baseline [16, 24, 28, 29]. The RAPID-axSpA [30] and ESTHER [16] studies both found that there was not a significant difference in treatment response between AS and nr-axSpA patients. As demonstrated in these studies as well as in our current study, the severity of symptoms, the disease activity, and the clinical characteristics of AS and nr-axSpA are similar between these two groups of patients.

While this study provides a comparison of AS and nr-axSpA patients in the USA and the results are primarily consistent with findings in different geographical areas, some limitations of this analysis should still be considered. There could be the potential for bias based on the recruitment strategy since rheumatologists voluntarily participating in this study selected ten consecutive consulting patients with axSpA. These patients may not be representative of axSpA patients in the USA that are not being treated by a rheumatologist. Additionally, rheumatologists were not required to include axSpA patients who fulfilled the formal classification criteria or clinical test results, so misclassification could exist. In the USA, at the time of this survey, there were no approved treatments for nr-axSpA, and therefore some patients may have been mislabeled as AS in order to get the treatment as recommended by their rheumatologist. Despite these limitations, this study provides evidence that the clinical characteristics, symptomology, quality of life, and disease status of AS and nr-axSpA patients in the USA are similar. The shared characteristics of these AS and nr-axSpA patients are consistent with the prevalent opinion that AS and nr-axSpA are two subtypes on the spectrum of axSpA. AS and nr-axSpA patients face the same burden and need the same level of access to targeted advanced treatment.
